# Structural and functional echocardiographic characteristics in women with preeclampsia–eclampsia: a comparative study in northwestern Tanzania

**DOI:** 10.3389/fmed.2026.1901122

**Published:** 2026-09-16

**Authors:** Fatma Mohammed, Eva Mujuni, Fridolin Mujuni, Richard Kiritta, Edgar Ndaboine

**Affiliations:** 1 Department of Obstetrics and Gynecology, Abdulla Mzee Hospital, Pemba, Zanzibar, Tanzania; 2 Department of Internal Medicine (Cardiology), Bugando Medical Centre, Catholic University of Health and Allied Sciences, Mwanza, Tanzania; 3 Department of Obstetrics and Gynecology, Bugando Medical Centre, Catholic University of Health and Allied Sciences, Mwanza, Tanzania

**Keywords:** diastolic dysfunction, echocardiography, hypertensive disorders of pregnancy, left ventricular function, preeclampsia–eclampsia, structural cardiac abnormalities, Tanzania

## Abstract

**Background:**

Preeclampsia–eclampsia is a multisystem hypertensive disorder associated with structural and functional cardiac alterations. However, echocardiographic data from low-resource settings remain limited. This study compared structural and functional echocardiographic characteristics between women with preeclampsia–eclampsia and normotensive pregnant women attending Bugando Medical Centre in northwestern Tanzania.

**Methods:**

A comparative cross-sectional study was conducted among 273 women who were at least 28 weeks pregnant or within 10 days postpartum between January 2022 and January 2023. Participants comprised 135 women with preeclampsia–eclampsia and 138 normotensive controls. Transthoracic echocardiography was performed to assess cardiac structure and function. The primary outcomes were composite structural and functional echocardiographic abnormalities. Multivariable logistic regression was used to evaluate the association between blood pressure severity and echocardiographic abnormalities after adjustment for age, body mass index, parity, referral status, and timing of echocardiographic assessment.

**Results:**

Composite structural echocardiographic abnormalities were significantly more common among women with preeclampsia–eclampsia than among normotensive women (55.6% vs. 26.8%, *p* < 0.001), as were composite functional abnormalities (40.0% vs. 23.9%, *p* = 0.004). Women with preeclampsia–eclampsia had significantly greater interventricular septal thickness, left ventricular posterior wall thickness, and relative wall thickness, while left ventricular internal dimensions did not differ significantly between groups. Reduced left ventricular ejection fraction (11.9% vs. 5.1%, *p* = 0.044) and diastolic dysfunction (31.1% vs. 20.3%, *p* = 0.041) were more prevalent in the preeclampsia–eclampsia group. Increasing systolic and diastolic blood pressure severity remained independently associated with composite structural abnormalities after multivariable adjustment, whereas no independent association was observed with the composite functional outcome.

**Conclusion:**

Women with preeclampsia–eclampsia had a significantly higher prevalence of structural and functional echocardiographic abnormalities than normotensive women. Structural abnormalities were more strongly related to increasing blood pressure severity than functional abnormalities, suggesting that hypertensive load is an important determinant of maternal cardiac structural adaptation. Prospective longitudinal studies are needed to determine the persistence and prognostic significance of these abnormalities after pregnancy.

## Introduction

Preeclampsia–eclampsia (PE-E) is a multisystem hypertensive disorder of pregnancy and remains a leading cause of maternal and perinatal morbidity and mortality worldwide. Recent estimates indicate that hypertensive disorders complicate approximately 4%–5% of pregnancies globally and are responsible for a substantial proportion of maternal deaths, particularly in low- and middle-income countries (1, 2). In sub-Saharan Africa, the burden is disproportionately high due to delayed diagnosis, limited access to specialized care, and referral delays. In Tanzania, studies conducted at tertiary centers such as Bugando Medical Centre have reported significant maternal morbidity and mortality associated with preeclampsia/eclampsia, underscoring the continued public health importance of PE-E in this setting (3–5).

Normal pregnancy is characterized by marked cardiovascular adaptations, including increased cardiac output, plasma volume expansion, and reduced systemic vascular resistance, which are essential for maintaining adequate uteroplacental perfusion (6). However, in PE-E, these physiological adaptations are disrupted by abnormal placentation, endothelial dysfunction, and increased vascular resistance, resulting in a maladaptive cardiovascular state. This altered hemodynamic environment leads to increased cardiac afterload and may trigger echocardiographic structural and functional alterations in the myocardium (7).

Recent evidence demonstrates that PE-E is associated with significant cardiac remodeling, including increased left ventricular mass, concentric hypertrophy, and impaired diastolic function (8, 9). Although left ventricular systolic function is often preserved, subtle functional impairment can be detected using echocardiographic techniques, particularly in severe disease (7). Furthermore, these cardiac alterations may persist beyond pregnancy and are associated with an increased long-term risk of cardiovascular disease, including hypertension and heart failure (5, 10, 11).

Transthoracic echocardiography is a non-invasive and widely available imaging modality that provides detailed assessment of cardiac structure and function. It plays a crucial role in detecting early myocardial changes, evaluating disease severity, and guiding clinical management in hypertensive disorders of pregnancy (2, 12). Despite its clinical utility, echocardiographic evaluation is not routinely incorporated into the management of PE-E in many low-resource settings, and data describing cardiac changes in these populations remain limited.

In Tanzania, and particularly at Bugando Medical Centre, there is limited contemporary evidence describing structural and functional cardiac alterations among women with PE-E. Understanding these changes is essential for improving risk stratification, optimizing management, and identifying women at risk of long-term cardiovascular complications. Therefore, this study aimed to compare structural and functional cardiac characteristics between women with preeclampsia–eclampsia and normotensive pregnant women attending Bugando Medical Centre.

## Methods

### Study design and setting

This comparative cross-sectional study was conducted between January 2022 and January 2023 in the Departments of Obstetrics and Gynecology and Cardiology at Bugando Medical Centre, Mwanza, Tanzania. Bugando Medical Centre is a tertiary referral and teaching hospital serving the northwestern regions of Tanzania.

### Study population and eligibility criteria

The study included pregnant women attending antenatal clinics, admitted to obstetric wards, or within 10 days postpartum at Bugando Medical Centre. Women were eligible if they had a gestational age of at least 28 weeks or were within 10 days after delivery and provided informed consent to participate in the study. Women with chronic hypertension, diabetes mellitus, pre-existing cardiovascular disease, or those in active labour at the time of assessment were excluded.

### Study groups and diagnostic definitions

Participants were classified into two study groups: women with preeclampsia–eclampsia and normotensive women. Preeclampsia was defined as new-onset hypertension, with systolic blood pressure ≥140 mmHg and/or diastolic blood pressure ≥90 mmHg after 28 weeks of gestation, accompanied by proteinuria or evidence of maternal organ dysfunction. Preeclampsia with severe features was defined by systolic blood pressure ≥160 mmHg and/or diastolic blood pressure ≥110 mmHg, or the presence of thrombocytopenia, impaired liver function, renal insufficiency, pulmonary oedema, persistent neurological symptoms, or visual disturbance. Eclampsia was defined as the occurrence of generalized seizures in a woman with preeclampsia when other causes of seizures had been excluded. Normotensive participants had systolic blood pressure <140 mmHg and diastolic blood pressure <90 mmHg without proteinuria or clinical evidence of preeclampsia.

### Blood-pressure measurement

Blood pressure was measured using an automated upper-arm blood-pressure monitor (Omron HEM-7120). Measurements were obtained with the participant seated or resting, with the arm supported at heart level. For participants with suspected hypertension, blood pressure was measured on two occasions at least four hours apart, unless urgent antihypertensive treatment was required. Urinary protein was assessed using URINOX-10 urine reagent strips. Relevant laboratory findings, including platelet count, serum creatinine, and liver transaminases, were obtained from the participants' clinical records.

### Sample size estimation

The sample size was calculated using the Kish–Leslie formula for cross-sectional studies, based on a previously reported prevalence of cardiac abnormalities among women with preeclampsia–eclampsia (13). A minimum sample size of 113 participants per group was obtained. To increase the power of the study and account for potential non-response, a 20% adjustment was applied, resulting in a total sample size of 273 participants.

### Sampling and recruitment

Eligible participants were recruited consecutively using a convenience sampling approach until the required sample size was achieved in each study group. Women with preeclampsia–eclampsia were recruited primarily from the obstetric wards and antenatal clinic. Normotensive participants were recruited from the same clinical areas during the same study period to ensure that both groups originated from the same underlying hospital population.

### Echocardiographic assessment

All participants underwent transthoracic echocardiography using a General Electric (GE) Vivid IQ ultrasound system equipped with a 3SC phased-array cardiac transducer (frequency range 1.5–3.6 MHz). Examinations were performed by an experienced cardiologist with participants positioned in the left lateral decubitus position. Standard parasternal long-axis, parasternal short-axis, and apical two-, three-, and four-chamber views were obtained using standard transthoracic echocardiographic techniques.

Two-dimensional and M-mode measurements obtained from the parasternal long-axis view were used to assess left atrial dimension, left ventricular internal diameter at end-diastole (LVIDd), left ventricular internal diameter at end-systole (LVIDs), interventricular septal thickness in diastole (IVSd), and left ventricular posterior wall thickness in diastole (LVPWd). Fractional shortening was derived from LVIDd and LVIDs. Relative wall thickness (RWT) was calculated using the end-diastolic ventricular dimension as 2×LVPWd/LVIDd. Left ventricular ejection fraction was estimated using the Teichholz method from M-mode-guided parasternal long-axis measurements, and an ejection fraction <50% was classified as reduced systolic function.

Left ventricular diastolic function was assessed using pulsed-wave Doppler recordings of mitral inflow at the tips of the mitral valve leaflets. Peak early diastolic filling velocity (E wave), peak atrial filling velocity (A wave), the E/A ratio, and deceleration time were assessed. Tissue Doppler imaging was used to measure early diastolic mitral annular velocity (e′), and the E/e′ ratio was used as an estimate of left ventricular filling pressure. Diastolic dysfunction was classified according to the Doppler and tissue Doppler criteria specified in the study protocol.

### Operational definitions

#### Structural echocardiographic abnormality

Structural echocardiographic abnormality was defined as the presence of at least one of the following: abnormal left ventricular internal diameter at end-diastole (LVIDd), abnormal interventricular septal thickness in diastole (IVSd), or abnormal left ventricular posterior wall thickness in diastole (LVPWd). Measurements were classified using the predefined reference ranges specified in the study protocol, with LVIDd considered normal between 33 and 55 mm and IVSd and LVPWd considered normal between 6 and 11 mm.

#### Functional echocardiographic abnormality

Functional echocardiographic abnormality was defined as the presence of reduced left ventricular ejection fraction below 50% and/or abnormal left ventricular diastolic function.

#### Timing of echocardiographic assessment

Echocardiography was performed either during pregnancy or within 10 days postpartum. Timing of assessment was categorized as antenatal at ≤34 weeks' gestation, antenatal at >34 weeks' gestation, or postpartum. Of the 273 participants, 56 (20.5%) underwent echocardiographic assessment antenatally at ≤34 weeks, 113 (41.4%) at >34 weeks, and 104 (38.1%) during the postpartum period. Because cardiovascular physiology differs between pregnancy and the early postpartum period, timing of echocardiographic assessment was included as a prespecified covariate in the multivariable logistic regression models to account for potential hemodynamic differences.

### Data collection

Sociodemographic and clinical information was collected using a structured questionnaire and participants' medical records. Variables collected included age, gestational age, parity, body mass index, referral status, obstetric characteristics, blood pressure measurements, laboratory findings, and treatment history where applicable. Echocardiographic data were obtained from standardized transthoracic examinations performed according to the study protocol, as described above.

### Data management and statistical analysis

Data were entered, cleaned, and analyzed using Stata version 13 (StataCorp, College Station, TX, USA). Categorical variables were summarized as frequencies and percentages, whereas continuous variables were assessed for normality using the Shapiro–Wilk test and graphical methods, and summarized as means ± standard deviations (SD) or medians with interquartile ranges (IQR), as appropriate. Comparisons between the preeclampsia–eclampsia and normotensive groups were performed using the chi-square test or Fisher's exact test for categorical variables, and Welch's two-sample *t*-test or the Mann–Whitney *U* test for normally and non-normally distributed continuous variables, respectively.

Composite structural and functional echocardiographic abnormalities were derived from their predefined component variables as described above. RWT was analyzed as a continuous descriptive echocardiographic parameter and was not included in the composite structural outcome. Logistic regression analysis was performed to evaluate the association between blood pressure severity and echocardiographic abnormalities. Both crude and adjusted odds ratios (ORs) with 95% confidence intervals (CIs) were reported. Multivariable models were adjusted for age, body mass index, parity, referral status, and timing of echocardiographic assessment (antenatal ≤34 weeks, antenatal >34 weeks, or postpartum). Statistical significance was defined as a two-sided *p* value <0.05.

### Ethical considerations

Ethical approval to conduct the study was obtained from the CUHAS/BMC Joint Ethical Review Committee (Ref No: CREC/517/2022). Written informed consent was obtained from all participants prior to enrollment. The study was conducted in accordance with relevant ethical guidelines and regulations, and confidentiality of participant information was strictly maintained.

## Results

A total of 273 pregnant women were enrolled in the study. Of these, 138 (50.6%) were normotensive and 135 (49.4%) had preeclampsia–eclampsia (PE-E). Among PE-E cases, 116 (42.5%) had severe preeclampsia, 10 (3.7%) had eclampsia, and 9 (3.3%) had preeclampsia without severe features.

### Sociodemographic and clinical characteristics

The baseline sociodemographic and clinical characteristics of the study participants are presented in Table 1. Women with preeclampsia–eclampsia were more frequently referred from other health facilities than normotensive women (52.6% vs. 39.1%, *p* = 0.035). The distribution of the timing of echocardiographic assessment (antenatal ≤34 weeks, antenatal >34 weeks, and postpartum) also differed significantly between the groups (*p* = 0.002). No significant differences were observed in age, residence, education level, marital status, occupation, parity, or body mass index.

**Table 1:** Baseline sociodemographic and obstetric characteristics of the study participants.

Variable	Normotensive (*n* = 138)	PE–E (*n* = 135)	*P* value
Age (years), mean ± SD	30.8 ± 5.1	30.2 ± 6.0	0.355
Age category			0.590
≤35 years	111 (80.4)	104 (77.0)	
>35 years	27 (19.6)	31 (23.0)	
Residence			0.792
Urban	89 (64.5)	84 (62.2)	
Rural	49 (35.5)	51 (37.8)	
Education			1.000
Formal education	127 (92.0)	124 (91.9)	
No formal education	11 (8.0)	11 (8.1)	
Marital status			0.519
Married	125 (90.6)	118 (87.4)	
Not married	13 (9.4)	17 (12.6)	
Occupation			0.443
Employed	92 (66.7)	83 (61.5)	
Not employed	46 (33.3)	52 (38.5)	
Admission source			0.035
Referral	54 (39.1)	71 (52.6)	
Self-referral	84 (60.9)	64 (47.4)	
Parity			0.056
Multiparous	110 (79.7)	93 (68.9)	
Nulliparous/Primiparous	28 (20.3)	42 (31.1)	
BMI (kg/m^2^), mean ± SD	28.1 ± 5.0	28.9 ± 5.7	0.232
BMI category			0.445
Normal	38 (27.5)	37 (27.4)	
Overweight	58 (42.0)	48 (35.6)	
Obese	42 (30.4)	50 (37.0)	
Timing of echocardiographic assessment			0.002
≤34 weeks gestation	26 (18.8)	30 (22.2)	
>34 weeks gestation	71 (51.4)	42 (31.1)	
Postpartum (≤10 days)	41 (29.7)	63 (46.7)	

Values are presented as *n* (%) unless otherwise stated. Continuous variables are presented as mean ± standard deviation. *P*-values were calculated using the chi-square test for categorical variables and Welch's *t*-test for continuous variables.

### Structural versus functional findings of echocardiographic measurements

Comparison of continuous echocardiographic measurements demonstrated significant differences in several structural parameters between the study groups (Table 2). Women with preeclampsia–eclampsia had significantly greater left atrial diameter, interventricular septal thickness, left ventricular posterior wall thickness, and relative wall thickness than normotensive women. In contrast, left ventricular internal dimensions in both diastole and systole, fractional shortening, left ventricular ejection fraction, and mitral E/A ratio did not differ significantly between the groups.

**Table 2:** Comparison of continuous echocardiographic measurements between women with preeclampsia–eclampsia and normotensive women.

Echocardiographic parameter	Normotensive (*n* = 138), Median (IQR)	PE–E (*n* = 135), Median (IQR)	*P* value^a^
Left atrial diameter (mm)	31.3 (28.5–35.9)	34.0 (29.8–37.4)	0.026
Left ventricular internal diameter in diastole (LVIDd, mm)	44.2 (38.8–49.1)	46.0 (40.3–49.9)	0.424
Left ventricular internal diameter in systole (LVIDs, mm)	30.0 (26.1–33.4)	30.1 (26.8–35.3)	0.343
Interventricular septal thickness in diastole (IVSd, mm)	9.1 (7.9–10.2)	10.6 (9.3–12.2)	<0.001
Left ventricular posterior wall thickness in diastole (LVPWd, mm)	9.0 (8.1–10.1)	10.2 (9.6–12.5)	<0.001
Relative wall thickness	0.417 (0.342–0.509)	0.466 (0.399–0.589)	<0.001
Fractional shortening (%)	36.4 (32.0–39.2)	34.3 (31.0–39.0)	0.178
Left ventricular ejection fraction (%)	66.0 (60.0–69.7)	63.2 (58.0–69.9)	0.235
Mitral E/A ratio	1.27 (0.82–1.46)	1.27 (0.87–1.52)	0.392

Data are presented as median [interquartile range (IQR)]. Relative wall thickness (RWT) was calculated as 2×LVPWd/LVIDd. LVIDd, left ventricular internal diameter at end-diastole; LVIDs, left ventricular internal diameter at end-systole; IVSd, interventricular septal thickness in diastole; LVPWd, left ventricular posterior wall thickness in diastole; PE–E, preeclampsia–eclampsia.

a *P* values were obtained using the Mann–Whitney *U* test because one or both study groups did not meet the normality assumption.

### Abnormal structural and functional echocardiographic findings

The prevalence of clinically abnormal structural and functional echocardiographic findings was compared between women with preeclampsia–eclampsia and normotensive women (Table 3). Women with preeclampsia–eclampsia had significantly higher frequencies of abnormal interventricular septal thickness, abnormal left ventricular posterior wall thickness, reduced left ventricular ejection fraction, and diastolic dysfunction. In contrast, the prevalence of abnormal LVIDd and left atrial enlargement did not differ significantly between the two groups.

**Table 3:** Prevalence of clinically abnormal structural and functional echocardiographic findings among women with preeclampsia–eclampsia and normotensive women.

Echocardiographic finding	Normotensive (*n* = 138), *n* (%)	PE–E (*n* = 135), *n* (%)	*P* value
Structural abnormalities			
Left atrial enlargement	4 (2.9)	5 (3.7)	0.944
Abnormal LVIDd (<33 or >55 mm)	23 (16.7)	26 (19.3)	0.577
Abnormal IVSd	16 (11.6)	53 (39.3)	<0.001
Abnormal LVPWd	11 (8.0)	55 (40.7)	<0.001
Functional abnormalities			
Reduced left ventricular ejection fraction (<50%)	7 (5.1)	16 (11.9)	0.044
Diastolic dysfunction	28 (20.3)	42 (31.1)	0.041

Values are presented as *n* (%). Between-group comparisons were performed using the chi-square test or Fisher's exact test, as appropriate. Structural abnormalities were classified using the predefined reference ranges specified in the study protocol: LVIDd 33–55 mm, IVSd 6–11 mm, and LVPWd 6–11 mm. LVIDd values outside the predefined range (<33 or >55 mm) were classified as abnormal. Left ventricular ejection fraction <50% was classified as reduced systolic function, while diastolic dysfunction was classified according to the predefined Doppler and tissue Doppler criteria. LVIDd, left ventricular internal diameter at end-diastole; IVSd, interventricular septal thickness in diastole; LVPWd, left ventricular posterior wall thickness in diastole; PE–E, preeclampsia–eclampsia.

### Composite structural and functional echocardiographic abnormalities

To summarize the overall burden of echocardiographic abnormalities, composite structural and functional outcomes were constructed from the predefined component variables. Women with preeclampsia–eclampsia had a significantly higher prevalence of both composite structural and functional echocardiographic abnormalities compared with normotensive women. The comparison of these primary study outcomes is presented in Table 4.

**Table 4:** Composite structural and functional echocardiographic abnormalities.

Outcome	Normotensive (*n* = 138), *n* (%)	PE–E (*n* = 135), *n* (%)	*P* value
Composite structural echocardiographic abnormality	37 (26.8)	75 (55.6)	<0.001
Composite functional echocardiographic abnormality	33 (23.9)	54 (40.0)	0.004

Composite structural echocardiographic abnormality was defined as the presence of at least one abnormal LVIDd, IVSd, or LVPWd measurement. Composite functional echocardiographic abnormality was defined as left ventricular ejection fraction <50% and/or abnormal diastolic function. Values are presented as *n* (%), and between-group comparisons were performed using the chi-square test. LVIDd, left ventricular internal diameter at end-diastole; IVSd, interventricular septal thickness in diastole; LVPWd, left ventricular posterior wall thickness in diastole; PE–E, preeclampsia–eclampsia.

### Association between blood pressure severity and echocardiographic abnormalities

In both crude and adjusted analyses, increasing systolic and diastolic blood pressure severity was significantly associated with composite structural echocardiographic abnormalities (Table 5). After adjustment for age, body mass index, parity, referral status, and timing of echocardiographic assessment, systolic blood pressure of 140–159 mmHg and ≥160 mmHg was associated with 2.58-fold and 7.79-fold higher odds of structural abnormalities, respectively. Similarly, diastolic blood pressure of 90–109 mmHg and ≥110 mmHg was associated with 2.50-fold and 7.28-fold higher adjusted odds of structural abnormalities, respectively. In contrast, no statistically significant independent association was observed between systolic or diastolic blood pressure severity and the composite functional outcome after multivariable adjustment.

**Table 5:** Crude and adjusted associations between blood pressure severity and composite structural and functional echocardiographic abnormalities.

Outcome/predictor	Category	Crude OR (95% CI)	*P* value	Adjusted OR (95% CI)	*P* value
Composite structural echocardiographic abnormality					
Systolic blood pressure	<140 mmHg	1.00 (Reference)	—	1.00 (Reference)	—
	140–159 mmHg	2.64 (1.44–4.84)	0.002	2.58 (1.39–4.81)	0.003
	≥160 mmHg	8.65 (4.06–18.46)	<0.001	7.79 (3.59–16.93)	<0.001
Diastolic blood pressure	<90 mmHg	1.00 (Reference)	—	1.00 (Reference)	—
	90–109 mmHg	2.61 (1.51–4.51)	<0.001	2.50 (1.43–4.38)	0.001
	≥110 mmHg	7.89 (2.99–20.85)	<0.001	7.28 (2.70–19.58)	<0.001
Composite functional echocardiographic abnormality					
Systolic blood pressure	<140 mmHg	1.00 (Reference)	—	1.00 (Reference)	—
	140–159 mmHg	1.73 (0.94–3.19)	0.078	1.53 (0.81–2.89)	0.190
	≥160 mmHg	1.32 (0.66–2.65)	0.427	1.42 (0.68–2.97)	0.349
Diastolic blood pressure	<90 mmHg	1.00 (Reference)	—	1.00 (Reference)	—
	90–109 mmHg	1.89 (1.08–3.30)	0.025	1.72 (0.96–3.07)	0.067
	≥110 mmHg	1.19 (0.48–2.92)	0.712	1.30 (0.51–3.34)	0.579

Crude odds ratios were obtained from univariable logistic regression models. Adjusted odds ratios were obtained from multivariable logistic regression models including age, body mass index, parity, referral status, and timing of echocardiographic assessment (antenatal ≤34 weeks, antenatal >34 weeks, or postpartum). Systolic and diastolic blood pressure categories were modeled separately to avoid collinearity. Composite structural echocardiographic abnormality was defined as at least one abnormal LVIDd, IVSd, or LVPWd measurement. Composite functional echocardiographic abnormality was defined as left ventricular ejection fraction <50% and/or abnormal diastolic function. OR, odds ratio; CI, confidence interval.

## Discussion

This study demonstrated that women with preeclampsia–eclampsia had a significantly higher burden of both structural and functional echocardiographic abnormalities than normotensive women. Women with preeclampsia–eclampsia exhibited significantly greater interventricular septal thickness, left ventricular posterior wall thickness, and relative wall thickness, whereas left ventricular internal dimensions did not differ significantly between the groups. These findings suggest that the observed structural differences were predominantly characterized by increased myocardial wall thickness rather than ventricular chamber dilatation. In addition, composite functional echocardiographic abnormalities, including reduced left ventricular ejection fraction and diastolic dysfunction, were significantly more frequent among women with preeclampsia–eclampsia. Furthermore, increasing systolic and diastolic blood pressure severity was independently associated with structural echocardiographic abnormalities after adjustment for potential confounding factors. These findings support the concept that preeclampsia–eclampsia is associated with measurable cardiovascular alterations involving both ventricular structure and function, with structural changes appearing to be more strongly related to hypertension severity than functional impairment (14, 15).

The significantly greater interventricular septal thickness, left ventricular posterior wall thickness, and relative wall thickness observed among women with preeclampsia–eclampsia are consistent with the haemodynamic adaptations associated with hypertensive disorders of pregnancy. In contrast, left ventricular internal dimensions did not differ significantly between the groups, suggesting that the predominant structural changes were related to myocardial wall thickening rather than ventricular chamber dilatation. Increased systemic vascular resistance and elevated afterload impose chronic pressure overload on the left ventricle, leading to compensatory myocardial hypertrophy characterized by thickening of the interventricular septum and posterior wall in an attempt to preserve cardiac output. These structural alterations are considered adaptive responses to increased cardiac workload during pregnancy rather than evidence of irreversible myocardial damage (14, 15).

Similar findings have been reported by Valensise et al., who demonstrated increased left ventricular wall thickness and concentric ventricular adaptation among women with preeclampsia, and by Bokslag et al., who concluded that persistent pressure overload contributes to structural cardiac changes that may extend beyond pregnancy (16, 17). The present findings therefore support growing evidence that preeclampsia–eclampsia is associated with significant structural cardiac alterations, emphasizing the importance of early recognition and optimal blood pressure control to reduce myocardial stress and potentially limit long-term cardiovascular sequelae.

In addition to the observed structural alterations, this study demonstrated a significantly higher prevalence of composite functional echocardiographic abnormalities among women with preeclampsia–eclampsia compared with normotensive women. This difference was primarily driven by a higher prevalence of reduced left ventricular ejection fraction and diastolic dysfunction in the preeclampsia–eclampsia group. These findings suggest that myocardial functional impairment may accompany the structural adaptations associated with hypertensive disorders of pregnancy, particularly among women with more severe disease. Elevated systemic vascular resistance, increased myocardial wall stress, endothelial dysfunction, and impaired myocardial relaxation have all been implicated in the development of ventricular dysfunction in preeclampsia–eclampsia (7, 15). Similar observations have been reported by Yerlikaya-Schatten et al. and Jiang et al., who demonstrated impaired myocardial relaxation and diastolic dysfunction in women with preeclampsia, with more pronounced abnormalities among those with severe disease, supporting the adverse effects of sustained pressure overload on myocardial performance (7, 18).

Although the prevalence of reduced left ventricular ejection fraction and diastolic dysfunction was significantly higher among women with preeclampsia–eclampsia, the continuous measurements of left ventricular ejection fraction and mitral E/A ratio did not differ significantly between the two groups. This apparent discrepancy likely reflects the fact that most participants maintained preserved systolic function despite the presence of hypertensive disease, resulting in similar median values for these continuous parameters. However, a greater proportion of women with preeclampsia–eclampsia crossed clinically established diagnostic thresholds for abnormal systolic and diastolic function, leading to significant differences in the categorical analyses. This finding highlights the importance of interpreting echocardiographic measurements within clinically validated reference ranges rather than relying solely on comparisons of central tendency. Similar findings have been reported in recent studies demonstrating that women with preeclampsia frequently maintain preserved left ventricular ejection fraction despite evidence of impaired diastolic function and subclinical myocardial dysfunction, particularly in severe disease, indicating that conventional systolic indices alone may underestimate cardiovascular involvement (7, 18–20).

An important finding of this study was the independent association between increasing blood pressure severity and composite structural echocardiographic abnormalities. In contrast to structural abnormalities, composite functional abnormalities were not independently associated with systolic or diastolic blood pressure severity after adjustment for potential confounders, suggesting that functional impairment may be influenced by factors beyond blood pressure severity alone. This finding supports the concept that the severity of hypertension is a major determinant of cardiac structural adaptation during preeclampsia–eclampsia. Persistent elevation of systemic vascular resistance increases left ventricular afterload, resulting in compensatory myocardial hypertrophy and ventricular wall thickening to maintain stroke volume and cardiac output. The graded relationship observed in this study suggests that progressive blood pressure elevation is accompanied by increasing myocardial structural changes, underscoring the importance of prompt recognition and effective control of severe hypertension during pregnancy to minimize cardiovascular complications (7, 15, 21).

The findings of this study have important clinical implications for the management of women with preeclampsia–eclampsia, particularly in low-resource settings. The high prevalence of structural and functional echocardiographic abnormalities observed among women with preeclampsia–eclampsia suggests that cardiovascular involvement may occur before the development of overt clinical heart failure. Echocardiographic assessment may therefore provide valuable information for identifying women with significant cardiac involvement, especially those with severe hypertension, persistent symptoms, or clinical suspicion of cardiovascular compromise. However, given the cross-sectional design of this study, the present findings do not support routine echocardiographic screening for all women with preeclampsia–eclampsia. Instead, echocardiography should be considered as an adjunctive diagnostic tool in selected high-risk patients while further prospective studies evaluate its role in improving maternal outcomes and predicting long-term cardiovascular risk (15, 21, 22).

### Strengths and limitations

This study has several strengths. To our knowledge, it is among the first studies from Tanzania to comprehensively compare both structural and functional echocardiographic characteristics between women with preeclampsia–eclampsia and normotensive women using standardized echocardiographic assessment. The inclusion of both antenatal and early postpartum participants reflects the spectrum of patients encountered in routine clinical practice at a tertiary referral hospital, and the use of multivariable regression analysis enabled adjustment for important maternal characteristics, including age, body mass index, parity, referral status, and timing of echocardiographic assessment.

Nevertheless, several limitations should be acknowledged. The cross-sectional design precludes establishing temporal or causal relationships between preeclampsia–eclampsia and the observed cardiac abnormalities. As a single-centre study conducted at a tertiary referral hospital, the findings may not be fully generalizable to lower-level healthcare facilities or the broader obstetric population. In addition, comprehensive contemporary indices of cardiac remodeling, including left ventricular mass index, left atrial volume index, and global longitudinal strain, were not assessed; therefore, the study provides evidence of structural and functional echocardiographic abnormalities rather than a complete characterization of cardiac remodeling. Finally, long-term postpartum follow-up was not performed, preventing assessment of whether the observed abnormalities resolved after delivery or persisted as markers of future cardiovascular disease.

## Conclusion

Women with preeclampsia–eclampsia had a significantly higher prevalence of both structural and functional echocardiographic abnormalities than normotensive pregnant women. Structural abnormalities were characterized predominantly by increased interventricular septal thickness, left ventricular posterior wall thickness, and relative wall thickness, while left ventricular internal dimensions did not differ significantly between the groups. Functional abnormalities included a higher prevalence of reduced left ventricular ejection fraction and diastolic dysfunction. Increasing systolic and diastolic blood pressure severity was independently associated with structural echocardiographic abnormalities, highlighting the impact of hypertension on maternal cardiac adaptation. These findings emphasize the importance of recognizing cardiovascular involvement in women with preeclampsia–eclampsia, particularly those with severe hypertension. Although routine echocardiographic screening cannot be recommended based on this cross-sectional study alone, targeted echocardiographic assessment in selected high-risk women may facilitate early identification of cardiac involvement and support individualized clinical management. Prospective longitudinal studies are needed to determine the persistence of these abnormalities after pregnancy and their implications for long-term maternal cardiovascular health.

## Data Availability

The raw data supporting the conclusions of this article will be made available by the authors, without undue reservation.
